# The effect of phytoplankton properties on the ingestion of marine snow by *Calanus pacificus*

**DOI:** 10.1093/plankt/fbab074

**Published:** 2021-11-12

**Authors:** Grace F Cawley, Moira Décima, Andrea Mast, Jennifer C Prairie

**Affiliations:** DEPARTMENT OF ENVIRONMENTAL AND OCEAN SCIENCES, UNIVERSITY OF SAN DIEGO, 5998 ALCALA PARK, SAN DIEGO, CA 92110-80091, USA; SCRIPPS INSTITUTION OF OCEANOGRAPHY, UNIVERSITY OF CALIFORNIA, SAN DIEGO, 9500 GILMAN DR., LA JOLLA, CA 92093-0227, USA; SCRIPPS INSTITUTION OF OCEANOGRAPHY, UNIVERSITY OF CALIFORNIA, SAN DIEGO, 9500 GILMAN DR., LA JOLLA, CA 92093-0227, USA; NATIONAL INSTITUTE OF WATER AND ATMOSPHERIC RESEARCH, WELLINGTON 6021, New Zealand; DEPARTMENT OF MARINE AFFAIRS, DALHOUSIE UNIVERSITY, 6299 SOUTH ST., HALIFAX, NOVA SCOTIA B2H 4R2, Canada; DEPARTMENT OF ENVIRONMENTAL AND OCEAN SCIENCES, UNIVERSITY OF SAN DIEGO, 5998 ALCALA PARK, SAN DIEGO, CA 92110-80091, USA

**Keywords:** marine snow, zooplankton, biological pump, trophic dynamics

## Abstract

Marine snow, formed through the aggregation of phytoplankton and other organic matter, can be consumed by various types of zooplankton, affecting both planktonic trophic dynamics and the export of carbon to depth. This study focuses on how two factors—phytoplankton growth phase and species—affect copepod feeding on marine snow. To do this, we conducted a series of grazing experiments using gut pigment and stable isotope methods to quantify the ingestion of the copepod, *Calanus pacificus*, on both marine snow aggregates and individual phytoplankton. Results demonstrate that marine snow can represent a substantial food source for copepods, comparable to rates on individual phytoplankton. Moreover, we found that both the overall ingestion and the relative ingestion of aggregates vs. individual phytoplankton depended on phytoplankton growth phase for experiments conducted with the diatom *Thalassiosira weissflogii*. Although copepods consumed aggregates composed of *Skeletonema marinoi* at similar rates as those composed of *T. weissflogii*, no effect of growth phase was observed for *S. marinoi*. These findings suggest that marine snow can be an important source of nutrition for copepods, but that its role in planktonic food webs may differ depending on the phytoplankton community composition and the stage of phytoplankton blooms.

## INTRODUCTION

Marine snow refers to aggregates that form in the ocean from phytoplankton, fecal pellets and other organic and inorganic matter (Alldredge and Silver, 1988). These aggregates play an important role in the carbon cycle since they sink significantly faster than individual phytoplankton (Iversen and Ploug, 2010; McDonnell and Buesseler, 2010). Many types of zooplankton, including copepods, can interact with these sinking aggregates, thus impacting carbon cycling and export (Turner, 2004). Many field observations of zooplankton associated with marine snow suggest that aggregates may be a substantial food source in addition to phytoplankton (Kiørboe, 2000; Möller *et al.,* 2012; Steinberg *et al.,* 1994). For example, using SCUBA to collect marine snow, Green and Dagg (1997) observed a variety of zooplankton in association with aggregates, including various copepod species such as *Oncaea spp.* and *Microsetella norvegica* that have been shown to specialize in the consumption of marine snow (Koski *et al.,* 2020). Shanks and Walters (1997) also observed copepods associated with aggregates in the field and, in laboratory experiments, used a vertical flume to further investigate interactions between copepods and marine snow aggregates. Gut content analysis has been used to confirm the consumption of marine snow by many different types of zooplankton (Bochdansky and Herndl, 1992; Dagg, 1993; Uttal and Buck, 1996; Wilson and Steinberg, 2010), including multiple species of copepods (Shanks and Edmondson, 1990; Van der Jagt *et al.,* 2020).

Our somewhat limited understanding of interactions between zooplankton and marine snow can be attributed to the fragile nature of marine snow, which cannot be sampled by traditional field methods. Observations of these interactions *in situ* have been bolstered by the advancement of imaging systems (Möller *et al.,* 2012). Further challenges with working with marine snow in the lab have provided barriers to experimentally quantifying ingestion of marine snow by zooplankton. Dilling *et al.* (1998) noted issues with many classical methods for quantifying ingestion when applied to marine snow but was able to measure the consumption of field-collected marine snow by *Calanus pacificus* using fecal pellet production. In one set of experiments, they were able to quantify the ingestion rate of *Euphausia pacifica* on marine snow through changes in particulate organic carbon concentration but acknowledged that high abundances of marine snow needed to be used because of the sensitivity in taking these measurements. Dilling and Brzezinski (2004) carried out grazing experiments with *E. pacifica* and *C. pacificus* in tanks with both individual phytoplankton and marine snow, which were labeled with different isotopic tracers. This study showed that marine snow is a viable food choice even when other alternatives are present; however, absolute ingestion rates were not quantified. Koski *et al.* (2017) similarly compared the consumption of aggregates vs. dispersed phytoplankton but in incubations where copepods were offered one of the two food sources; the ingestion rate on aggregates, which was quantified using fecal pellet production and respiration rates, depended on the copepod type. These studies have provided important insight into trophic interactions between zooplankton and aggregates and highlight some of the many complex factors that may affect these relationships.

Interactions between zooplankton and marine snow can have an impact on the pelagic food web by providing an alternative food source for copepods. Aggregation of phytoplankton cells may transform particles into a larger, more manageable size for ingestion by size-selective grazers (Frost, 1972; Hansen *et al.,* 1994). In this way, the formation of marine snow can allow phytoplankton that are too small to be eaten by some zooplankton to become newly available as a food source, creating a sort of trophic shortcut (Lampitt *et al.,* 1993; Stukel *et al.,* 2014). This may help explain the observation of *Synechococcus* in mesozooplankton fecal pellets in a pico-plankton dominated region (Stukel *et al.,* 2013), since *Synechococcus* has been shown to form aggregates readily (Cruz and Neuer, 2019). Along with affecting trophic dynamics, zooplankton ingestion of marine snow aggregates also impacts the biological pump in multiple ways. Even if zooplankton are not directly feeding on the aggregates, fragmentation of the particles can occur when zooplankton interact with them (Dilling and Alldredge, 2000; Goldthwait *et al.,* 2004; Kiko *et al.,* 2017; Kiørboe, 2001). This fragmentation will result in changes in the size and density of the marine snow particles, which will then alter their sinking rate (Gibbs, 1985; Prairie *et al.,* 2019), thus decreasing the efficiency of the biological pump. In addition, zooplankton repackage marine snow aggregates into dense fecal pellets (Shanks and Edmondson, 1990; Turner and Ferrante, 1979), which generally sink at faster rates than marine snow (Bruland and Silver, 1981). This effect of repackaging may be particularly important for certain species of copepods that are known to colonize these particles and for which marine snow makes up a substantial portion of their nutrition (Green and Dagg, 1997; Koski *et al.,* 2020).

Although many studies have examined zooplankton, including copepods, foraging on marine snow, there is not a lot known on how different properties of marine snow may impact consumption by zooplankton. Since copepods are known to select prey based on many factors that can include size, taxonomy and growth phase (Kiørboe, 2011), it is likely that the ingestion of marine snow by copepods may also depend on the physical and biological characteristics of the aggregates. For example, marine snow can vary in size and composition based on the different species and physiology of the phytoplankton present and other variables (Alldredge and Gotschalk, 1988; Engel *et al.,* 2009; Thornton and Thake, 1998; Yamada *et al.,* 2013). Properties of marine snow have also been shown to depend on the amount of TEP (transparent exopolymer particles) produced, which is the sticky matrix produced by phytoplankton and bacteria that acts like a glue helping with the aggregation process (Alldredge *et al.,* 1998; Passow, 2002). Phytoplankton, specifically diatoms, also develop different physiological characteristics with age (De Troch *et al.,* 2012), and previous studies have shown that zooplankton can demonstrate a food preference based on phytoplankton growth phase (Barofsky *et al.,* 2010; Long and Hay, 2006). Given this, ingestion of marine snow may also depend on the age of the phytoplankton from which they are formed, particularly since Prairie *et al.* (2019) showed that phytoplankton growth phase affected the TEP production and density of marine snow. Despite this, no study has specifically looked at the effect of phytoplankton growth phase on the ingestion of marine snow aggregates by zooplankton, although Dilling *et al.* (1998) observed copepod foraging on aged marine snow from the ocean. Examining how factors such as phytoplankton species and growth phase affect copepod ingestion of marine snow is important to predicting how these interactions could impact carbon export temporally and spatially in the ocean, as well as understanding zooplankton reliance on alternate food sources such as marine snow.

In this study, we investigated how specific properties of phytoplankton impact copepod foraging on marine snow aggregates. With a series of lab experiments, the ingestion of the copepod *C. pacificus* on marine snow was quantified using both gut pigment analysis and stable isotope analysis. Ingestion of marine snow and dispersed phytoplankton were compared for different phytoplankton growth phases and for two different species of phytoplankton.

## METHODS

During the summer of 2018 and the autumn of 2019, six experiments were conducted to investigate the effect of phytoplankton properties on the ingestion rate of *C. pacificus* (Table I). All experiments included two or three growth phases and phytoplankton as a food source presented in both dispersed (i.e. as individual cells) and aggregated form. Each growth phase included three treatments: (i) a control, in which copepods were placed in tanks with filtered seawater and no food source, (ii) a phytoplankton treatment, in which copepods were placed in tanks with dispersed phytoplankton as a food source, and (iii) an aggregate treatment, in which copepods were placed in tanks with aggregates as a food source. Ingestion rate was quantified in these experiments using two methods: gut pigment analysis and stable isotope analysis.

**Table I:** Description of the copepod grazing experiments, including duration that each culture was grown for each growth phase, dates of the experiment for each growth phase, sample size for gut pigment analysis and stable isotope analysis (given for control, phytoplankton and aggregate singular, respectively, with the sample sizes combined for the two replicate tanks), and the phytoplankton species used in each experiment. Growth phases are abbreviated as Early Exp (for early exponential), Late Exp (for late exponential) and Late Stat (for late stationary)

Exp.	Growth phase	Experiment date	Sample size for gut pigment	Sample size for stable isotope	Phytoplankton species
1	Early Exp (5 Days)	6/13/18	20, 18, 19	8, 8, 8	*T. weissflogii*
	Late Exp (11 Days)	6/19/18	16, 15, 17	7, 7, 7	
	Late Stat (17 Days)	6/25/18	6, 8, 7	N/A	
2	Early Exp (5 Days)	7/25/18	20, 19, 19	8, 8, 8	*T. weissflogii*
	Late Exp (11 Days)	7/31/18	19, 19, 20	8, 8, 8	
	Late Stat (17 Days)	8/6/18	9, 18, 15	4, 8, 8	
3	Early Exp (5 Days)	9/23/19	19, 19, 20	10, 10, 10	*T. weissflogii*
	Late Exp (12 Days)	9/30/19	20, 19, 17	10, 10, 10	
4	Early Exp (5 Days)	10/7/19	18, 18, 18	10, 10, 10	*S. marinoi*
	Late Exp (12 Days)	10/14/19	19, 18, 18	10, 10, 10	
5	Early Exp (5 Days)	11/11/19	19, 17, 17	10, 10, 10	*T. weissflogii*
	Late Exp (12 Days)	11/18/19	17, 20, 18	10, 10, 10	
6	Early Exp (5 Days)	11/25/19	20, 18, 20	10, 10, 10	*S. marinoi*
	Late Exp (12 Days)	12/9/19	17, 16, 20	10, 10, 10	

### Copepod collection

*Calanus pacificus* was collected using a small boat off the coast of La Jolla, CA (32° 51.720’ N, 117° 16.816’ W) 5–20 days before each experiment with a 333-μm mesh plankton net (0.5-m diameter mouth). The average sea surface temperature for collection dates ranged between 18 and 23°C. Contents of each tow were diluted and chilled, and samples were sorted for individuals of the species *C. pacificus* (copepodite V and adult female stages). Copepods were maintained with regular water changes in an incubator at 18°C in the dark. When being kept before experiments, copepods were fed a mixed diet of phytoplankton as to not develop a preference: *Thalassiosira weissflogii* and haptophytes (*Tisochrysis sp.* and *Pavlova sp.*) for the 2018 experiments and *T. weissflogii* and *Skeletonema marinoi* for the 2019 experiments. Copepods were acclimated to room temperature (~21°C) and starved 24 hours prior to the experiment to avoid residual food in their gut (Dam and Peterson, 1988). Beakers were wrapped in aluminum foil during acclimation period to keep copepods in the dark.

### Phytoplankton cultures and aggregate formation

**Fig. 1:**
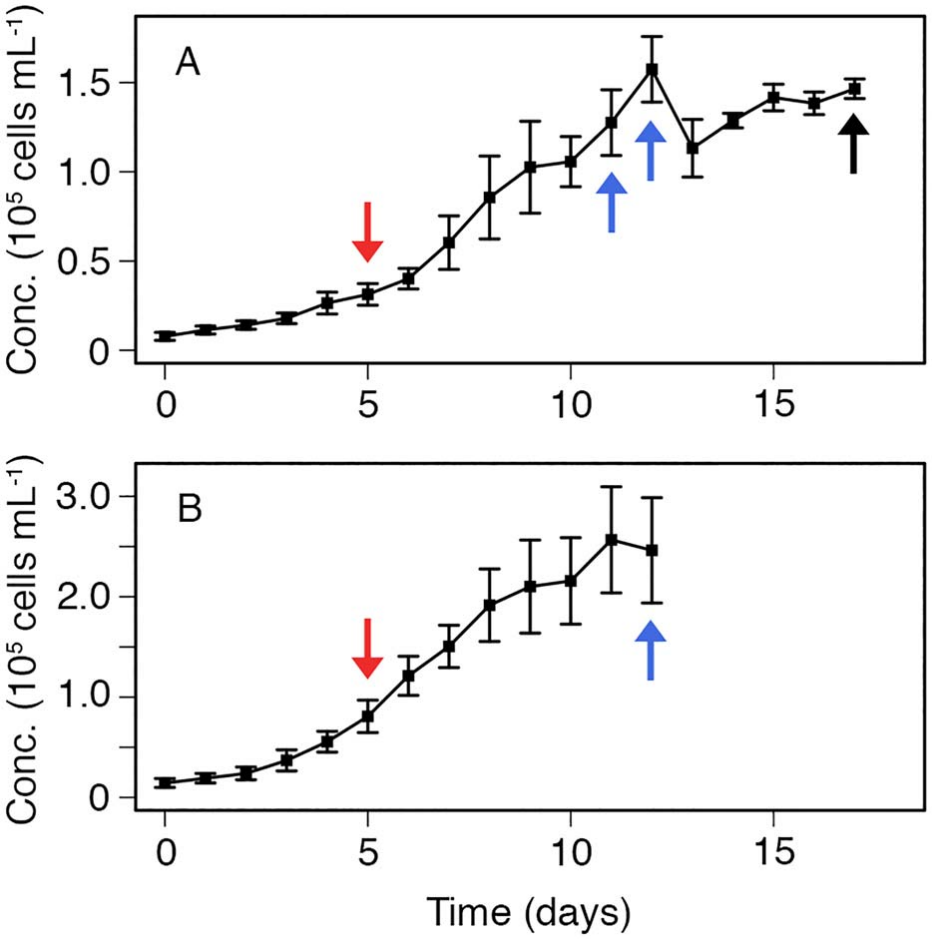
(A) Cell concentration (cells/mL) of the culture of *T. weissflogii* vs. time (in days after the culture was started) averaged over Experiments 2, 3 and 5. (B) Cell concentration (cells/mL) of the culture of *S. marinoi* vs. time (in days after the culture was started) averaged over Experiments 4 and 6. Error bars represent standard deviation. Red arrows indicate time that cultures were stopped for early exponential growth phase, blue arrows indicate time that cultures were stopped for late exponential growth phase (this differed between Experiments 1/2 and 3/5 for *T. weissflogii* experiments), and black arrow indicates time that cultures were stopped for late stationary growth phase (which was only done for Experiments 1 and 2).

Prior to each experiment, non-axenic phytoplankton cultures of the species *T. weissflogii* (Experiments 1, 2, 3 and 5) or *S. marinoi* (Experiments 4 and 6) were started in 2 L flasks (Table I). All cultures were grown in f/2 media at room temperature (~21°C) under a 12:12 hour LED light:dark cycle. Experiments 1 and 2 were carried out for three different growth phases (corresponding to early exponential, late exponential and late stationary stages of the phytoplankton growth curve), while Experiments 3 through 6 were carried out with just the first two growth phases. For each growth phase, two cultures were started: one to be used for the dispersed phytoplankton treatment and one to be used for the aggregate treatment. Cultures for the early exponential growth phase were grown for 5 days, cultures for the late exponential growth phase were grown for 11 days (Exp. 1 and 2) or 12 days (Exp. 3–6) and cultures for the late stationary growth phase were grown for 17 days (Table I). In all cases, cultures for the aggregate treatment were started 3 days earlier than the cultures for the phytoplankton treatment to account for the 3-day period used for rolling the culture to form marine snow (see description below). The cell concentration of each phytoplankton culture was measured every day on a particle counter (Multisizer 3, Beckman Coulter Counter) to track phytoplankton growth over time (Fig. 1), with the exception of Experiment 1, in which phytoplankton growth was tracked through daily measurements of *in vivo* fluorescence.

To carry out ingestion rate measurements using stable isotope analysis, 1.7 mL (or 12.75 mL in Exp. 1) of a ^15^N nitrate solution (73.5 μM ^15^N-potassium nitrate salt in DI water) was added to each phytoplankton culture 2–5 days before each culture was to be stopped. Right before adding the ^15^N nitrate solution to each culture, 25 mL of the culture was filtered onto GF/F filters in triplicate, to be used as initial measurements of naturally occurring ^15^N concentrations in the phytoplankton cultures. Filters were frozen in a −20°C freezer until all isotope samples were ready to be packed for analysis.

After the cultures for the aggregate treatments grew for their allotted time (Table I), they were diluted and added to two cylindrical acrylic tanks, each with a volume of 550 mL. Due to the difference in sizes between the two species, which were estimated from the Coulter Counter (*T. weissflogii* had an average diameter of ~10 μm and *S. marinoi* had an average diameter of ~8 μm), the cultures of *T. weissflogii* and *S. marinoi* were diluted to 20,000 and 39,000 cells mL^−1^, respectively, to allow for a roughly equivalent concentration by cell volume between the two species. These tanks were incubated on a roller table and allowed to rotate at a speed of 4.6 rpm for 3 days to form aggregates. This method has been widely used previously to form aggregates (Dilling and Brzezinski, 2004; Prairie *et al.,* 2013; Shanks and Edmondson, 1989). Tanks were incubated on the roller table in the dark to ensure that no further growth of the phytoplankton culture occurred.

### Grazing experiments

For each treatment, two replicate cylindrical tanks were used, each with a volume of 2200 mL. For the aggregate treatment, aggregates formed in the 550-mL tanks were transferred to the experimental tank along with the seawater in which they were formed; the rest of the volume of the experimental tank (total 2200 mL) was filled with filtered seawater. Since the entire contents of the 550-mL tank were transferred into the experimental tank (which was four times the volume), this resulted in an average cell concentration of 5000 cells mL^−1^ for *T. weissflogii* and 9750 cells mL^−1^ for *S. marinoi* in the experimental tanks for the aggregate treatments. This method may have resulted in some unaggregated phytoplankton in the aggregate treatments, but the presence of numerous visible aggregates in each experiment indicates that the concentration of unaggregated phytoplankton was a small fraction of what was present in the phytoplankton treatment. Before adding copepods, aggregates were photographed on a transparent mm-square grid sealed on the bottom of the cylindrical experimental tank to observe differences in the size and appearance of aggregates from different growth phases and experiments (photographs were not taken in Experiments 1 and 2). The phytoplankton treatment tanks were filled with individual phytoplankton from the culture grown for this treatment which was diluted to 5000 cells mL^−1^ for *T. weissflogii* and 9750 cells mL^−1^ for *S. marinoi*, such that the phytoplankton treatment tanks and the aggregate treatment tanks had equivalent cell concentrations. Control treatment tanks were filled with filtered seawater and had a small amount of the ^15^N nitrate solution added (between 87 and 483 μL) such that the final concentration of ^15^N in the control tanks was comparable to that in the phytoplankton and aggregate tanks for that experiment. For each replicate treatment tank, 30 copepods were added (except for the late stationary growth phase in Exp. 1 for which 16 copepods were added) and allowed to forage for an hour while the tank rotated at ~1 rpm in the dark.

Once the 1-hour incubation time had elapsed for each treatment tank, the tank was removed from the roller table and 40 mL of seltzer water was added to anesthetize the copepods and avoid regurgitation of gut contents. The copepods were removed from the cylindrical experimental tank with gentle suctioning of water onto a mesh sieve. For gut pigment analysis, two copepods were placed in 6–10 amber vials (depending on the total number of copepods recovered), which contained 3 mL of 90% acetone. For stable isotope analysis, five sets of two copepods were transferred into tin cups for each tank of each treatment (only 3–4 sets of two copepods were collected for Experiments 1 and 2).

### Gut pigment analysis

After copepods were transferred to amber vials, a sonicator was used at 40% amplitude for 5 seconds to break up the organisms and release their gut content into the acetone solution. In addition, the water from each experimental tank was evenly mixed, and three subsamples of 25 mL of tank water were filtered onto GF/F filters and placed into 5 mL of acetone. After a day in a −20°C freezer, the copepod and tank water samples were analyzed using a Trilogy Laboratory Fluorometer (Turner Designs) to measure the concentration of total pigment (combined chlorophyll-*a* and pheophytin) in the acetone solution. For the copepod samples, this represents the gut pigment content per copepod from the experiment (*G*, in units of μg pigment copepod^−1^) which was calculated using the equation (Dam and Peterson, 1988)

(1) \begin{equation*} G=\frac{K\ \left(\frac{r}{r-1}\right)\ \left({rR}_a-{R}_a\right)\ E}{n_s}, \end{equation*}

where *K* is the fluorometer calibration constant, *R_a_* represents the fluorescence reading after acidification, *r* is the acidification ratio, *E* is the volume in *L* of acetone used to extract chlorophyll and ${n}_s$ is the number of copepods per vial. To account for differences in fluorescence between growth phases and between phytoplankton and aggregates (since the aggregates were formed in the dark for 3 days), the fluorescence in the tank water in units of μg pigment L^−1^ (combined chlorophyll-*a* and pheophytin) was calculated from the fluorometer measurements of the tank water samples based on EPA method 445.0. These values were averaged for every treatment (separately for each experiment and growth phase), after removing any outliers (defined as any values greater than 3 interquartile ranges above the third quartile or any values less than 3 interquartile ranges below the first quartile). The average fluorescence per cell (*F*, in units of μg pigment cell^−1^) was then calculated for each treatment of every growth phase and experiment according to the following equation:

(2) \begin{equation*} F=\frac{{{Flt}}_{{w}}}{C\left(1-p\right)}, \end{equation*}

where ${{Flt}}_{{w}}$ is the average fluorescence in the tank water as described above, *C* is the initial phytoplankton concentration in the tank in cells L^−1^ (5,000,000 cells L^−1^ for *T. weissflogii* experiments and 9,750,000 cells L^−1^ for *S. marinoi* experiments) and *p* is the proportion of initial fluorescence that was consumed by copepods, calculated as

(3) \begin{equation*} p=\frac{n_t\ {G}_{\mathrm{ave}}}{n_t\ {G}_{\mathrm{ave}}+{V}_t{{Flt}}_{{w}}}, \end{equation*}

where ${n}_t$ is the number of copepods in each tank, ${G}_{\mathrm{ave}}$ is the average gut pigment content per copepod and ${V}_t$ is the volume of the experimental tank (2.2 L) (Supplementary Table SI). To directly compare results between the two methods, we calculated mass of food per copepod (${M}_{\mathrm{food}}$, in units of μgC copepod^−1^) using

(4) \begin{equation*} {M}_{\mathrm{Food}}=\left(\frac{G}{F}\right)\times{M}_{\mathrm{cell}}, \end{equation*}

where ${M}_{\mathrm{cell}}$ is the mass of carbon per cell as calculated by the relationship provided in Menden-Deuer and Lessard (2000) using the average cell diameters estimated from the Coulter Counter (10 μm for *T. weissflogii* and 8 μm for *S. marinoi*) and assuming the cells were spherical. Note that *F* could not be directly calculated for the control treatment (since *C* is equal to 0 cells L^−1^), and therefore, ingestion for these samples was calculated using the *F* value for the phytoplankton treatment from the same experiment.

### Stable isotope analysis

In addition to the copepods used in the grazing experiments, for each growth phase of each experiment five sets of two unfed copepods (which were starved alongside experimental copepods but not used in any treatment tank) were transferred into tin cups and were used to measure the natural concentration of ^15^N in copepods before being exposed to phytoplankton grown in the ^15^N nitrate solution. Right after grazing incubations were complete, subsamples of the remaining tank water for each treatment (three replicates of 250 mL) were each filtered onto GF/F filters and packed into a tin cup for ^15^N measurements of the food that was fed to the copepods in each treatment. The GF/F filters taken prior to adding the ^15^N nitrate solution to the cultures were also packed into tin cups to be analyzed. All samples were processed by UC Davis Stable Isotope Facility (with the exception of Experiment 1, in which samples were processed on an Isotope Ratio Mass Spectrometer at Scripps Institution of Oceanography).

From the raw stable isotope data, isotopic fraction of each copepod sample (${F}_s$) was calculated as (Verschoor *et al.,* 2005)

(5) \begin{equation*} {F}_s=\frac{R_S}{\left({R}_S+1\right)}, \end{equation*}

where ${R}_S$ is the isotopic ratio of the sample calculated as

(6) \begin{equation*} {R}_S=\left(\left(\frac{\delta 15N}{1000}\right)+1\right) {R}_R, \end{equation*}

where ${R}_R$ is the isotopic ratio of a reference standard (Sigman *et al.,* 2009) and $\delta$  ^15^N is the measure of the ratio of ^15^N to ^14^N that is provided in the raw data.

The mass of food per copepod (${M}_{\mathrm{food}}$) was calculated as

(7) \begin{equation*} {M}_{\mathrm{Food}}=\frac{M_{\mathrm{FedZoop}}{F}_{\mathrm{FedZoop}}-\left({F}_{\mathrm{Starved}} {M}_{\mathrm{Starved}}\right)}{F_{\mathrm{Food}}}, \end{equation*}

where ${M}_{\mathrm{FedZoop}}$ is the mass of carbon in the experimental copepod sample (as given in the stable isotope data) divided by the number of copepods, *F*_FedZoop_ and *F*_Starved_ are the isotopic fraction of the experimental copepods and the starved copepods, respectively (calculated using equation 5) and *F*_Food_ is the isotopic fraction of the food source (calculated using equation 5 but from the measurements of the GF/F filters taken from the remaining water of the feeding experiments for the phytoplankton and aggregate treatment, and from the measurements of the GF/F filters taken from the cultures before ^15^N was added for the control treatment). ${M}_{\mathrm{Starved}}$ represents the mass of the experimental copepods before they were fed; however, because this measurement could not be obtained, ${M}_{\mathrm{FedZoop}}$ was used in place of ${M}_{\mathrm{Starved}}$ in equation 7 (which assumes that the difference in mass before and after the copepods were fed is negligible). In cases where equation 7 resulted in a negative value for ${M}_{\mathrm{Food}}$ (which occurred for some samples in the control treatment when *F*_Starved_ was greater than ${F}_{\mathrm{FedZoop}}$), a value of 0 was used for ${M}_{\mathrm{Food}}$ instead.

### Estimation of ingestion rates

Ingestion rates were estimated from the food per copepod measurements described above by considering a range of gut passage times. Gut passage time was calculated based on the relationship provided in Dam and Peterson (1988) at 21°C is 16 minutes, although the relationship developed in that study was based on observations primarily in colder waters and does not hold well in higher temperatures. As an example, Dam *et al.* (1995) estimated the gut passage time at 26°C at the equator to be 28.5 minutes. Conservatively, we estimated gut passage time as equal to the incubation time, 1 hour. Thus, food per copepod data was converted into ingestion rates by multiplying by a constant that ranges from 1 to 3.75 hour^−1^.

Using the stable isotope copepod data, for which the mass of the samples is known, we calculated weight-specific ingestion rates. The average ingestion rate was normalized per copepod mass by dividing equation 7 by ${M}_{\mathrm{FedZoop}}$ (see Supplementary Table SII for values) and assuming 12 hours of grazing per day to convert to daily rates in order to represent natural behavior—e.g. diel vertical migration, Frost 1988. Respiration measurements were calculated using metabolic rate equations developed for marine zooplankton by Ikeda (1985).

### Data analysis

A two-way mixed ANOVA was run on the pooled data for experiments using each phytoplankton species (and data from each methodology separately) with growth phase and treatment as fixed effects and tank/experiment number as a random effect. For the *T. weissflogii* experiments, the ANOVA was only run on the first two growth phases since the late stationary growth phase was only done in Experiments 1 and 2. A Tukey–Kramer *post hoc* test was used to determine differences pairwise between the three treatments. A Mann–Whitney U-test was used to test for differences between the average food per copepod in *T. weissflogii* experiments and *S. marinoi* experiments for the different growth phases, treatments and methodologies.

To quantify aggregate size and shape, ImageJ was used to trace around all visible aggregates in the photographs taken before copepods were added to the experimental tanks. Area and perimeter were quantified for each aggregate, and circularity (a metric describing shape) was calculated as

(8) \begin{equation*} \mathrm{circularity}=\frac{4\ \pi\ \mathrm{area}}{{\mathrm{perimeter}}^2}, \end{equation*}

where a perfect circle will have a circularity value of 1 and circularity decreases to a minimum of 0 for highly non-circular shapes. Two-way mixed ANOVAs were run on the area and circularity data for each phytoplankton species with growth phase and experiment as fixed effects and tank number as a random effect.

## RESULTS

**Fig. 2:**
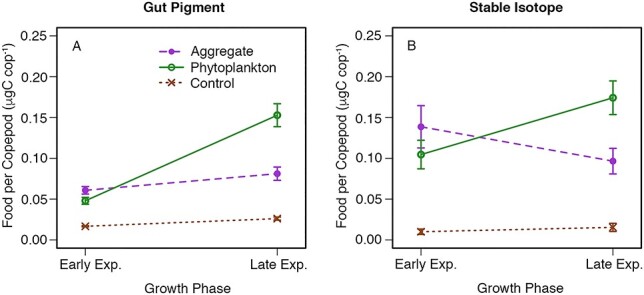
Interaction plots of food per copepod vs. growth phase (on *x*-axis) and treatment (shown in different colors and symbols) pooled for the four experiments using the phytoplankton species *T. weissflogii* for the two different methods: (A) gut pigment analysis and (B) stable isotope analysis. Error bars represent standard error. Two-way mixed-effect ANOVA tests for (A) show significant effect of treatment (*P* < 0.001), significant effect of growth phase (*P* < 0.001) and significant interaction effect (*P* < 0.001). Two-way mixed-effect ANOVA tests for (B) show significant effect of treatment (*P* < 0.001), no significant effect of growth phase (*P* > 0.05), and no significant interaction effect (*P* > 0.05).

For the experiments with *T. weissflogii*, there was no significant difference between the ingestion of aggregates and dispersed phytoplankton (as measured by gut pigment content) in the early exponential growth phase; however, in the late exponential growth phase, ingestion of dispersed *T. weissflogii* was significantly higher than that of aggregates (Fig. 2A, Table II). Results from the two-way mixed-effect ANOVA demonstrate a significant effect of treatment and growth phase, as well as a significant interaction effect. This indicates that growth phase not only affected the overall food per copepod, which was higher in late exponential compared with early exponential, but also affected the *relative* consumption of aggregates vs. phytoplankton. This pattern in the relative consumption of these food sources is echoed in the isotope data (Fig. 2B), with the ingestion of dispersed phytoplankton being higher in the late exponential growth phase; however, in this case, the interaction effect was not significant (Table II). These overall patterns are supported by the consistent results observed in each of the four experiments measuring ingestion with *T. weissflogii*.

**Table II:** *P*-values for Tukey–Kramer post-hoc pairwise comparisons of differences in mean food per copepod for pooled data for each phytoplankton species and growth phase as calculated from gut pigment data and stable isotope data (A = Aggregate, C = Control, P = Phytoplankton); asterisks indicate *P* < 0.05

Phytopl. species	Growth phase	Gut pigment	Stable isotope
*T. weissflogii*	Early exponential	A-C: *P* = 0.027^*^ A-P: *P* > 0.05 P-C: *P* > 0.05	A-C: *P* = 0.006^*^ A-P: *P* > 0.05 P-C: *P*> 0.05
	Late exponential	A-C: *P* = 0.010^*^ A-P: *P* < 0.001^*^ P-C: *P* < 0.001^^*^^	A-C: *P* > 0.05 A-P: *P* > 0.05 P-C: *P* = 0.002^^*^^
*S. marinoi*	Early exponential	A-C: *P* = 0.002^*^ A-P: *P* > 0.05 P-C: *P* = 0.038^^*^^	A-C: *P* > 0.05 A-P: *P* > 0.05 P-C: *P* = 0.026^^*^^
	Late exponential	A-C: *P* < 0.001^*^ A-P: *P* = 0.012^*^ P-C: *P* = 0.044^^*^^	A-C: *P* > 0.05 A-P: *P* > 0.05 P-C: *P* = 0.004^^*^^

Using gut pigment analysis, ingestion of aggregates was always roughly equal to the ingestion of dispersed phytoplankton in early exponential growth phase, but in late exponential growth phase, ingestion of dispersed phytoplankton increased such that it was higher than that of aggregates (Supplementary Fig. S1). The same pattern was also observed when using stable isotopes to measure ingestion, with the exception of the first experiment in which consumption of phytoplankton decreased from early to late exponential growth phase (Supplementary Fig. S1B). In the two experiments (Exp. 1 and Exp. 2) in which ingestion was also measured for late stationary growth phase, we observed a decrease in ingestion of dispersed phytoplankton relative to late exponential growth phase, such that the consumption of phytoplankton and aggregates was similar (Supplementary Fig. S1).

**Fig. 3:**
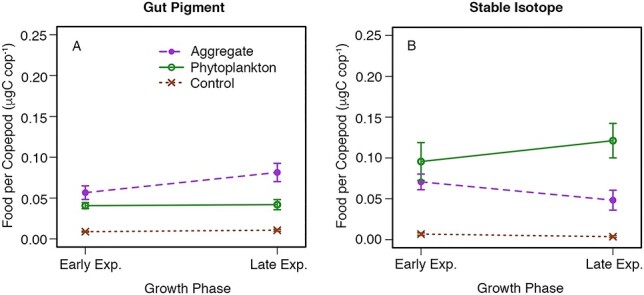
Interaction plots of food per copepod vs. growth phase (on *x*-axis) and treatment (shown in different colors and symbols) pooled for the two experiments using the phytoplankton species *S. marinoi* for the two different methods: (A) gut pigment analysis and (B) stable isotope analysis. Error bars represent standard error. Two-way mixed-effect ANOVA tests for (A) show significant effect of treatment (*P* < 0.001), no significant effect of growth phase (*P* > 0.05) and no significant interaction effect (*P* > 0.05). Two-way mixed-effect ANOVA tests for (B) show significant effect of treatment (*P* < 0.001), no significant effect of growth phase (*P* > 0.05) and no significant interaction effect (*P* > 0.05).

Ingestion on *S. marinoi* did not exhibit the same pattern as seen with *T. weissflogii*, with no effect of growth phase and no significant interaction effect (Fig. 3A). Mean food per copepod measured using gut pigment analysis was similar between growth phases with ingestion of aggregates higher than the ingestion of dispersed phytoplankton for both early exponential and late exponential growth phase (but with this difference being significant only in the latter growth phase) (Fig. 3A, Table II). Ingestion data for *S. marinoi* calculated using the stable isotope method also showed no effect of growth phase, although in this case the consumption of phytoplankton was higher, but not significantly different (Fig. 3B). Individual experiments showed qualitatively similar patterns for the gut pigment data (Supplementary Fig. S2A and C), but with some differences between the two experiments using the stable isotope method (Supplementary Fig. S2B and D).

Fluorescence values per cell for the phytoplankton treatment were greater than those for the aggregate treatment in 11 of the 14 growth-phase experimental trials (Supplementary Table SI). These differences were not large enough to substantially change the patterns for the experiments with *T. weissflogii* between raw gut pigment (Supplementary Fig. S3A) and food per copepod after it was corrected for this difference (Fig. 2A). With the pooled raw gut pigment data, there remained a significant effect of treatment, growth phase and interaction effect. However, the larger differences in the fluorescence values between phytoplankton and aggregates for the *S. marinoi* experiments resulted in differences between raw gut pigment (Supplementary Fig. S3B) and corrected food per copepod (Fig. 3A), with a significant effect of growth phase and a significant interaction effect observed for the raw gut pigment data.

**Fig. 4:**
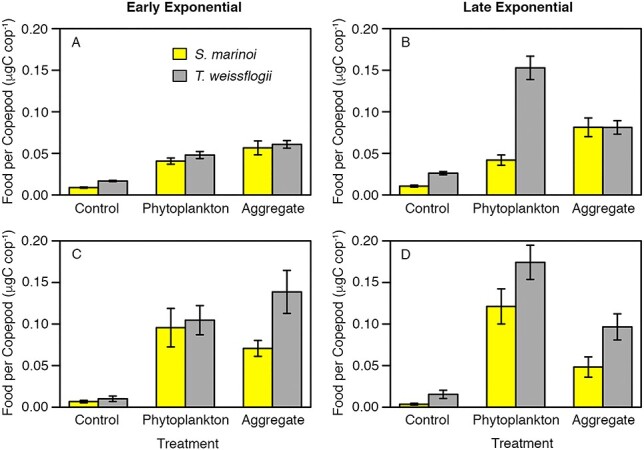
Bar graphs comparing the mean food per copepod calculated from gut pigment data between experiments conducted with *S. marinoi* (yellow) and *T. weissflogii* (gray) for early exponential (A) and late exponential (B) growth phases. (C and D) The same except for food per copepod calculated from stable isotope data between experiments. Error bars represent standard error.

In comparing ingestion on aggregates formed from the two different phytoplankton species, no significant difference was observed between the mean food per copepod of aggregates formed from *T. weissflogii* and *S. marinoi* for either growth phase based on gut pigment data (early exponential *P* > 0.05, late exponential growth phase *P* > 0.05) (Fig. 4A and B). Based on stable isotope data, mean food per copepod was higher in both growth phases for aggregates formed from *T. weissflogii* compared with aggregates formed from *S. marinoi*, but these differences were not significant (early exponential growth phase *P* > 0.05, late exponential growth phase *P* > 0.05) (Fig. 4C and D). The ingestion of dispersed phytoplankton calculated from gut pigment analysis was significantly higher for *T. weissflogii* in late exponential growth phase (*P* < 0.001) but was not statistically different between the two species in all other cases (gut pigment: early exponential growth phase *P* > 0.05; stable isotope: early exponential growth phase *P* > 0.05, late exponential growth phase *P* > 0.05).

**Fig. 5:**
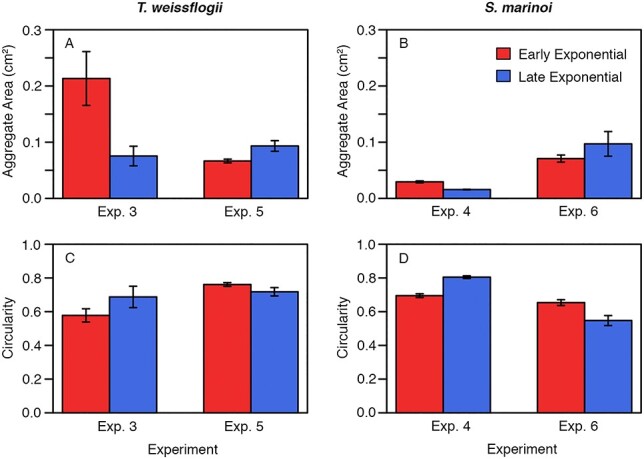
Bar graphs comparing the average area of aggregates between experiments (x-axis) and growth phase (colors) for *T. weissflogii* (A) and *S. marinoi* (B). (C and D) The same except for comparing the average circularity of aggregates. Error bars represent standard error. Two-way mixed-effect ANOVA tests show significant effect of experiment for panels A, B and D (for A, *P* = 0.004; for B *P* < 0.001; for C, *P* > 0.05 and for D, *P* = 0.030), but no significant effect of growth phase for any panel (for A, *P* > 0.05; for B, *P* > 0.05; for C, *P* > 0.05 and for D, *P* > 0.05).

**Fig. 6:**
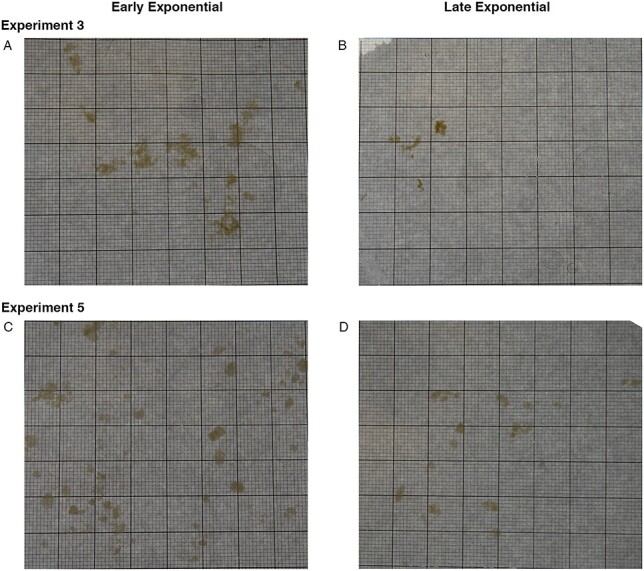
Images of *T. weissflogii* aggregates from one of the two aggregate treatment tanks for Experiment 3 early exponential growth phase (A), Experiment 3 late exponential growth phase (B), Experiment 5 early exponential growth phase (C) and Experiment 5 late exponential growth phase (D). Small square grids in images measure 1 mm^2^.

**Fig. 7:**
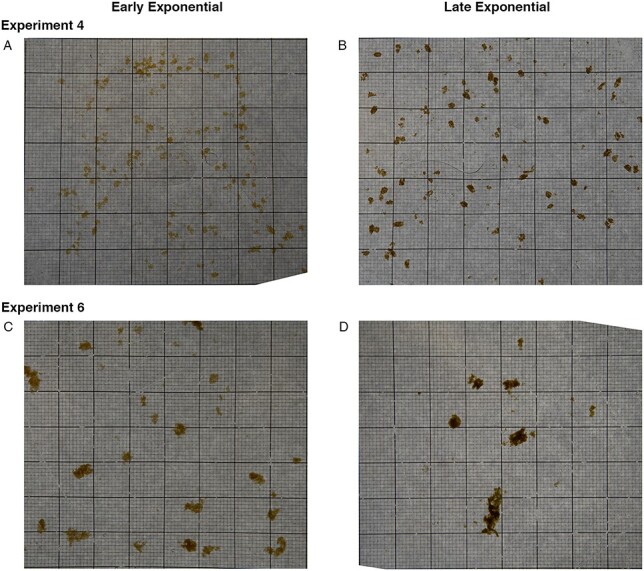
Images of *S. marinoi* aggregates from one of the two aggregate treatment tanks for Experiment 4 early exponential growth phase (A), Experiment 4 late exponential growth phase (B), Experiment 6 early exponential growth phase (C) and Experiment 6 late exponential growth phase (D). Small square grids in images measure 1 mm^2^.

Photographs of aggregates taken before each grazing experiment revealed differences in the size of aggregates between experiments for both *T. weissflogii* and *S. marinoi*, as well as significant differences in the shape of aggregates between experiments for *S. marinoi* (Fig. 5). However, neither species showed differences in either size or shape of aggregates between growth phases (Fig. 5). This variability can be observed in example photographs of aggregates from the experiments with *T. weissflogii* (Fig. 6) and *S. marinoi* (Fig. 7). In addition, the compactness of aggregates can be qualitatively observed from the color of the aggregates; *S. marinoi* aggregates in the early exponential growth phase for both experiments appeared less compact (as indicated by their lighter color) than their later growth phase counterparts (Fig. 7). In contrast, *T. weissflogii* did not show substantial differences in the color or compactness of aggregates between growth phases or experiments (Fig. 6).

Ingestion rates on *T. weissflogii* aggregates, calculated from food per copepod from gut pigment data, ranged from 0.061 to 0.228 μgC copepod^−1^ hour^−1^ for early exponential growth phase and 0.081 to 0.304 μgC copepod^−1^ hour^−1^ for late exponential growth phase. Similarly, aggregate ingestion rates calculated from gut pigment data on *S. marinoi* would be 0.057–0.212 μgC copepod^−1^ hour^−1^ for early exponential growth phase and 0.081–0.305 μgC copepod^−1^ hour^−1^ for late exponential growth phase. For the stable isotope data, aggregate ingestion rates for *T. weissflogii* were 0.139–0.520 μgC copepod^−1^ hour^−1^ for early exponential growth phase and 0.097–0.362 μgC copepod^−1^ hour^−1^ for late exponential growth phase, and for *S. marinoi* were 0.071–0.265 μgC copepod^−1^ hour^−1^ for early exponential growth phase and 0.048–0.181 μgC copepod^−1^ hour^−1^ for late exponential growth phase.

Using the stable isotope copepod data, the average ingestion rate normalized per copepod mass for our *T. weissflogii* experiments ranged from 0.0039 to 0.0246 μgC μgC^−1^ hour^−1^ for the dispersed phytoplankton treatment depending on growth phase and gut passage time, equivalent to the consumption of 4.7–29.5% of copepod body weight per day. In comparison, copepods in the aggregate treatment consumed 0.0035–0.0186 μgC μgC^−1^ hour^−1^, equivalent to 4.2–22.3% of body weight per day.

## DISCUSSION

Our study design, including two methodologies, multiple sets of experiments, and different growth phases and phytoplankton species, confirmed previous experiments suggesting active consumption of *C. pacificus* on phytoplankton aggregates (Dilling *et al.,* 1998). Our approach allowed us to additionally show that ingestion of both food types (marine snow and dispersed phytoplankton) was similar, yet both overall ingestion and relative ingestion on marine snow vs. dispersed phytoplankton depended on phytoplankton growth phase for experiments with *T. weissflogii* (Fig. 2). Utilization of two different species of phytoplankton further illustrated the diversity of factors that may affect the consumption of marine snow in the field, as grazing experiments with the smaller diatom *S. marinoi* did not show the same effect of growth phase as experiments with *T. weissflogii* (Fig. 3), although average ingestion of aggregates did not differ between the two species (Fig. 4).

The goal of the present work was to investigate the patterns of marine snow and phytoplankton consumption using two different methods, because quantifying ingestion of marine snow is notoriously difficult (Dilling *et al.,* 1998). We have structured our discussion below to address the strengths and limitations of our two methodological approaches, summarize our new understanding of aggregate consumption by copepods and address the implications of these results for plankton ecology.

### Comparison of gut pigment and stable isotope analyses for measuring ingestion of aggregates

Although many studies have demonstrated that copepods and other types of zooplankton consume marine snow (e.g. Dilling *et al.,* 1998; Steinberg *et al.,* 1994), quantifying the ingestion rate of marine snow by different organisms is challenging, since many methods used to measure the ingestion of phytoplankton may not be practical or accurate for grazing experiments with aggregates. For example, measuring ingestion through disappearance requires knowing the concentration of phytoplankton cells, which is not possible in irregularly shaped and fragile marine snow particles. Previous studies have quantified ingestion on aggregates using respiration rates and fecal pellet production (Dilling *et al.,* 1998; Koski *et al.,* 2017). In this study, we used two different methods commonly used in grazing experiments and adapted them so they could be applied to quantify the ingestion of marine snow aggregates in a way that is directly comparable to the ingestion of dispersed phytoplankton. In our experiments, the food per copepod calculated from these two independent methods was very close in magnitude (rarely differing by a factor of more than 2) and for *T. weissflogii* showed similar patterns with respect to treatment and growth phase, suggesting that both methodologies can provide consistent measurements of consumption of marine snow aggregates.

Our choice to compare two methodologies in our experiments necessitated that the number of copepods within our experimental tanks had to be split for the two analyses, with roughly two-thirds of the copepods being used for gut pigment analysis and the remaining one-third being used for stable isotope analysis. The smaller sample sizes for stable isotope analysis resulted in larger standard error for these data (Figs 2B and 3B), which was likely the reason that the same statistical patterns were not observed. High levels of individual variability in consumption rates, while not the focus of our study, have been reported from in-depth studies of *C. pacificus* in the field and lab (Karakoylu *et al.,* 2009; Karakoylu and Franks, 2012). Understanding the general species patterns that hold despite the large variability related to individual feeding thus is challenging, and statistically significant results (when present) indicate important patterns that are robust given that our sample size was low. In future studies, using a single methodology would allow for increased sample size, which would help further reveal relationships.

Although our study demonstrates that both gut pigment analysis and stable isotope analysis can be used to measure ingestion of marine snow, each method has advantages and limitations. Gut pigment analysis can also be used to measure marine snow consumption in the field (Möller *et al.,* 2012). However, gut pigment analysis is dependent on the fluorescence of individual cells, which we found can vary between dispersed phytoplankton and aggregates (Supplementary Table SI), and pigment destruction after gut passage has also been documented (Conover *et al.*, 1986). We were able to measure and account for differences in fluorescence per cell in our calculations of food per copepod, but this may be difficult or impossible when using this method in the field. Using stable isotope analysis for quantifying ingestion of marine snow requires adding an isotope as a tracer, and therefore this method may not be as easily adaptable as a shipboard technique. However, this method can be modified by using additional stable isotope tracers to separately quantify the ingestion of phytoplankton and aggregates to determine active selection between these two food sources, as was done by Dilling and Brzezinski (2004).

For both methodologies, gut passage time is important to take into account since both experiments measure food in the copepod at the end of the experiment. We chose to keep our incubations short (1 hour) to minimize this effect; however, since gut passage time was not directly measured, our results and primarily presented in terms of mass of food instead of as ingestion rates.

The average weight-specific ingestion rates for our *T. weissflogii* experiments (ranging between 4.2 and 29.5% of copepod body weight per day depending on treatment and gut passage time) are reasonable considering field estimates on the high end for female *C. pacificus* are 39% of body weight per day (Frost, 1972), and minimum requirements for respiration are ~10% of body weight per day at the temperature of our experiments (~20°C). Comparing to other studies can be hard because these rates are not always measured in the same way. Dilling *et al.* (1998) found that adult *C. pacificus* feeding on aggregates consume 0.3–1.8 μgC copepod^−1^ hour^−1^, which is at the high end of our estimates. However, their incubations were longer, done with natural marine snow, and a different method was used to quantify ingestion (fecal pellet production). Perhaps most importantly, since only adults were used in Dilling *et al.* (1998) (whereas our study had a mix of copepodite Vs and adult females), the weight-specific ingestion may be more similar. Thus, not only are the grazing rates measured in our experiments comparable for copepods feeding on phytoplankton and aggregates, but also, on average, they were sufficient to maintain basic metabolic needs suggesting that feeding rates were not substantially depressed within our laboratory experiments.

### Factors affecting ingestion of aggregates by copepods

Since aggregates are much lower in concentration than individual phytoplankton, it may be surprising that marine snow is readily consumed by copepods. Although aggregates are less likely to be happened upon by a grazer, these large particles represent a sort of patch of food (Kiørboe, 2001). If an individual copepod can find this large source of nutrition for relatively little energy cost because aggregates are abundant, it could be optimal to exploit this food source (DeMott, 1989; Pyke, 1984). Bacterial activity on marine snow as it sinks also creates a chemical trail that can be used by zooplankton to locate aggregates, making them more exploitable as a resource than if copepods relied solely on random encounters (Kiørboe, 2001; Lombard *et al.,* 2013). In situations when phytoplankton are present mostly in the dispersed form, the energy cost to find aggregates may be too high, resulting in the consumption of primarily individual phytoplankton. Though our experimental design did not allow us to test this hypothesis directly since copepods were not given a choice between dispersed and aggregated phytoplankton, a previous study by Dilling and Brzezinski (2004) found substantial consumption of aggregates by *C. pacificus* even in the presence of dispersed phytoplankton. Moreover, it is clear that the extent to which aggregates are consumed depends on the species of copepod. Koski *et al.* (2017) found that aggregates represented the primary food source for one harpacticoid and one poecilamastoid species of copepod, while the three species of calanoid copepods in their experiments—different from the one in this study—were not able to feed substantially on phytoplankton when it was in the aggregated form. Thus, the fact that we found comparable rates of ingestion for *C. pacificus* on both aggregates and dispersed phytoplankton is notable.

There is little known about differences in nutritional value of aggregates compared with individual phytoplankton. However, the presence of TEP, needed for the formation of aggregates, may play a role since copepods (specifically our study species *C. pacificus*) have been known to forage on these gel-like particles (Ling and Alldredge, 2003), although other studies have shown that TEP may deter grazing in some cases (Dutz *et al.,* 2005). Prieto *et al.* (2001) suggested that copepods could trigger a higher production of TEP when interacting with diatoms, creating a potential positive feedback loop in nutritional production that subsequently promotes aggregate formation.

The observed impact of growth phase on copepod ingestion may be a result of changes in phytoplankton physiology as the cultures grow and deplete nutrients to a point that induces stress. Some of these physiological responses could include changes to the biochemical composition of phytoplankton (Harrison *et al.,* 1990), as zooplankton have been shown to discriminate against dead cells in grazing experiments (Demott, 1988), and rolling aggregates in the dark for 3 days, potentially places these phytoplankton in a state of senescence and lowers their nutritional value (Harrison *et al.,* 1990). This could also explain the drop in consumption in the late stationary growth phase (measured in Experiments 1 and 2, Supplementary Fig. S1), which coincides with the cultures reaching a state of senescence (Kahl *et al.,* 2008).

In a similar way that phytoplankton characteristics such as size, presence of toxins, ability to form chains, and spines can deter grazing, variability in ingestion of marine snow based on phytoplankton growth phase and phytoplankton species may be a result of physical changes to aggregates (i.e. size, density and porosity). For example, aggregate density can depend on the growth phase of the phytoplankton cultures (Prairie *et al.,* 2019), potentially explaining our qualitative observations of the changes in the apparent compactness of *S. marinoi* aggregates between growth phases (Fig. 7). Both size and shape of marine snow aggregates can also vary based on the phytoplankton that are present (Li and Logan, 1995; Logan and Wilkinson, 1990). However, we observed overall consistent patterns between experiments of the same phytoplankton species—especially with *T. weissflogii—*despite the large inter-experiment variability in the size and shape of aggregates (Fig. 5); it is unclear whether these same patterns would hold in the ocean where the diversity of aggregate sizes is even greater—ranging from micrometers to centimeters in size (Alldredge and Silver, 1988).

Lastly, the bacterial community that grows alongside the phytoplankton likely varies depending on phytoplankton species and growth phase (Pinhassi *et al.,* 2004). These bacteria are not only present but often necessary in the marine snow formation process, including specifically for one of the phytoplankton species, *T. weissflogii*, used in this study (Gärdes *et al.,* 2011). These bacteria–phytoplankton interactions could introduce other factors affecting the consumption of marine snow by zooplankton (Mayor *et al.,* 2014).

### Significance to plankton ecology

Although phytoplankton have always been known to provide food for copepods and other grazers alike, more recently studies have introduced the notion that the aggregation of phytoplankton into marine snow could provide an additional nutritional pathway for copepods (e.g. Dilling and Brzezinski, 2004; Steinberg *et al.,* 1994). The results of this study further challenge the classical understanding of pelagic food webs that place phytoplankton (as individual cells) as the sole food source for herbivorous zooplankton, as demonstrated by ingestion of *C. pacificus* on marine snow aggregates being similar to that on dispersed phytoplankton. Thus, the aggregation of smaller phytoplankton into larger marine snow particles provides a trophic pathway that is often not considered in pelagic food web models.

Moreover, the observed effect of growth phase on the ingestion of marine snow by copepods suggests that the effects of these trophic interactions on carbon flux may be regulated by the seasonality of phytoplankton growth. For example, based on our results, earlier stages of blooms of *T. weissflogii* might experience higher relative ingestion of aggregates, affecting carbon export either positively through production of fecal pellets or negatively by inhibiting the direct flux of phytodetritus. Our findings demonstrate that understanding how different phytoplankton characteristics can affect the ingestion of both individual phytoplankton and aggregates is important for predicting zooplankton grazing during different times and phases of a phytoplankton bloom.

Our study emphasizes that understanding trophic dynamics requires knowledge of the dependencies on multiple factors, such as species composition and physiological state—both can affect grazing dynamics and trophic transfer. The inclusion of aggregate foraging behavior into biogeochemical modeling can improve predictions of carbon export flux by including an important interaction currently not included or well parameterized in carbon export flux models. The complexity of the ocean necessitates an understanding of how multiple simultaneous factors affect different interactions in the ocean to be able to predict the larger scale impacts of these interactions on global processes.

## CONCLUSIONS

Our study demonstrated that ingestion of aggregates by *C. pacificus* is comparable to their ingestion of individual phytoplankton, indicating that marine snow may represent an important additional food source. Furthermore, we found that the ingestion of aggregates by *C. pacificus* can vary depending on the growth phase of the phytoplankton from which the aggregates are formed, although this relationship in turn depends on phytoplankton species. Trophic interactions between copepods and marine snow are complex, making it challenging to estimate the net effect on pelagic food web dynamics and marine carbon cycling.

## DATA ARCHIVING

Data for this study are hosted by the Biological and Chemical Oceanography Data Management Office (BCO-DMO) and can be found at https://www.bco-dmo.org/project/699441.

## Supplementary Material

Cawley_SupplmentaryMaterial_fbab074
