# Supplementary material for: The effect of phytoplankton properties on the ingestion of marine snow by Calanus pacificus
Source: J Plankton Res. 2021 Nov 12;43(6):957–73. doi: 10.1093/plankt/fbab074 (PMC8632794; doi:10.1093/plankt/fbab074)
Supplement: Cawley_SupplmentaryMaterial_fbab074 [file cawley_supplmentarymaterial_fbab074.docx]

**SUPPLEMENTARY MATERIAL**

Supplementary Table SI. Average gut pigment ($G_{ave}$), average fluorescence in tanks post-experiment (${Fl}_{tw})$, proportion of fluorescence consumed by copepods (*p*), and fluorescence per cell (*F*) for each growth phase and treatment (with Phyto representing treatment with dispersed phytoplankton and Agg representing treatment with aggregates) of each experiment.

| Exp. | Growth Phase | Treatment | $G_{ave}$ (ng pigment cop^-1^) | ${Fl}_{tw}$ (μg pigment L^-1^) | *p* | *F* (10^-6^ μg pigment cell^-1^) |
| --- | --- | --- | --- | --- | --- | --- |
| 1 | Early Exp | Phyto | 5.60 | 14.86 | 0.0051 | 2.99 |
|  | Early Exp | Agg | 7.46 | 20.84 | 0.0049 | 4.19 |
|  | Late Exp | Phyto | 8.47 | 17.35 | 0.0066 | 3.49 |
|  | Late Exp | Agg | 5.92 | 18.61 | 0.0043 | 3.74 |
|  | Late Stat | Phyto | 4.43 | 12.43 | 0.0026 | 2.49 |
|  | Late Stat | Agg | 3.45 | 9.24 | 0.0027 | 1.85 |
| 2 | Early Exp | Phyto | 3.66 | 19.02 | 0.0026 | 3.81 |
|  | Early Exp | Agg | 4.15 | 11.74 | 0.0048 | 2.36 |
|  | Late Exp | Phyto | 12.90 | 13.28 | 0.0131 | 2.69 |
|  | Late Exp | Agg | 8.29 | 13.25 | 0.0085 | 2.67 |
|  | Late Stat | Phyto | 5.19 | 11.30 | 0.0062 | 2.27 |
|  | Late Stat | Agg | 4.33 | 8.50 | 0.0069 | 1.71 |
| 3 | Early Exp | Phyto | 2.04 | 17.63 | 0.0016 | 3.53 |
|  | Early Exp | Agg | 3.06 | 14.84 | 0.0028 | 2.98 |
|  | Late Exp | Phyto | 8.21 | 10.27 | 0.0108 | 2.08 |
|  | Late Exp | Agg | 3.24 | 13.55 | 0.0033 | 2.72 |
| 4 | Early Exp | Phyto | 3.28 | 26.34 | 0.0017 | 2.71 |
|  | Early Exp | Agg | 4.13 | 17.15 | 0.0033 | 1.76 |
|  | Late Exp | Phyto | 6.47 | 41.05 | 0.0021 | 4.22 |
|  | Late Exp | Agg | 7.70 | 35.80 | 0.0029 | 3.68 |
| 5 | Early Exp | Phyto | 2.72 | 17.75 | 0.0021 | 3.56 |
|  | Early Exp | Agg | 2.01 | 15.43 | 0.0018 | 3.09 |
|  | Late Exp | Phyto | 7.29 | 18.49 | 0.0053 | 3.72 |
|  | Late Exp | Agg | 3.18 | 16.39 | 0.0026 | 3.29 |
| 6 | Early Exp | Phyto | 4.27 | 22.77 | 0.0026 | 2.34 |
|  | Early Exp | Agg | 3.80 | 19.39 | 0.0027 | 1.99 |
|  | Late Exp | Phyto | 4.73 | 28.84 | 0.0022 | 2.96 |
|  | Late Exp | Agg | 7.99 | 19.95 | 0.0054 | 2.06 |

Supplementary Table SII. Average mass of carbon in the experimental copepod sample (as given in the stable isotope data) divided by the number of copepods ($M_{FedZoop}$) for each growth phase and treatment (with Phyto representing treatment with dispersed phytoplankton and Agg representing treatment with aggregates) averaged for the experiments with *T. weissflogii* and the experiments with *S. marinoi*.

| Exp. | Growth Phase | Treatment | $M_{FedZoop}$ (μgC cop^-1^) |
| --- | --- | --- | --- |
| Average for  *T. weissflogii* Experiments | Early Exp | Control | 29.5 |
|  | Early Exp | Phyto | 26.7 |
|  | Early Exp | Agg | 27.9 |
|  | Late Exp | Control | 26.4 |
|  | Late Exp | Phyto | 26.6 |
|  | Late Exp | Agg | 27.9 |
| Average for  *S. marinoi* Experiments | Early Exp | Control | 21.6 |
|  | Early Exp | Phyto | 19.9 |
|  | Early Exp | Agg | 31.8 |
|  | Late Exp | Control | 13.4 |
|  | Late Exp | Phyto | 17.5 |
|  | Late Exp | Agg | 22.0 |


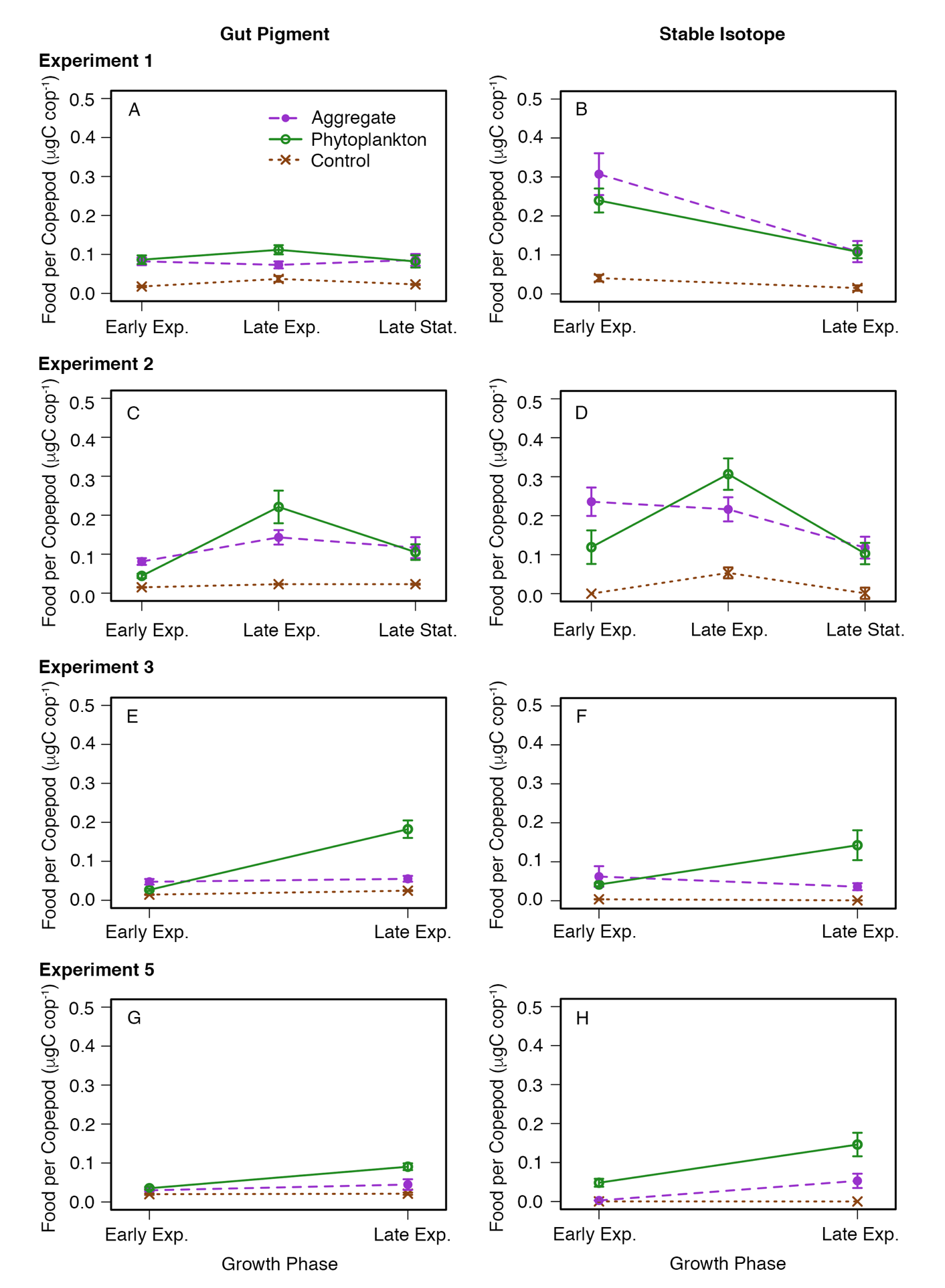


Supplementary Figure S1. Interaction plots of food per copepod versus growth phase (on x-axis) and treatment (shown in different colors and symbols) as calculated from gut pigment data (first column) and stable isotope analysis data (second column) for the four experiments using the phytoplankton species *T.weissflogii*: (A and B) Experiment 1, (C and D) Experiment 2, (E and F) Experiment 3, and (G and H) Experiment 5. Error bars represent standard error.


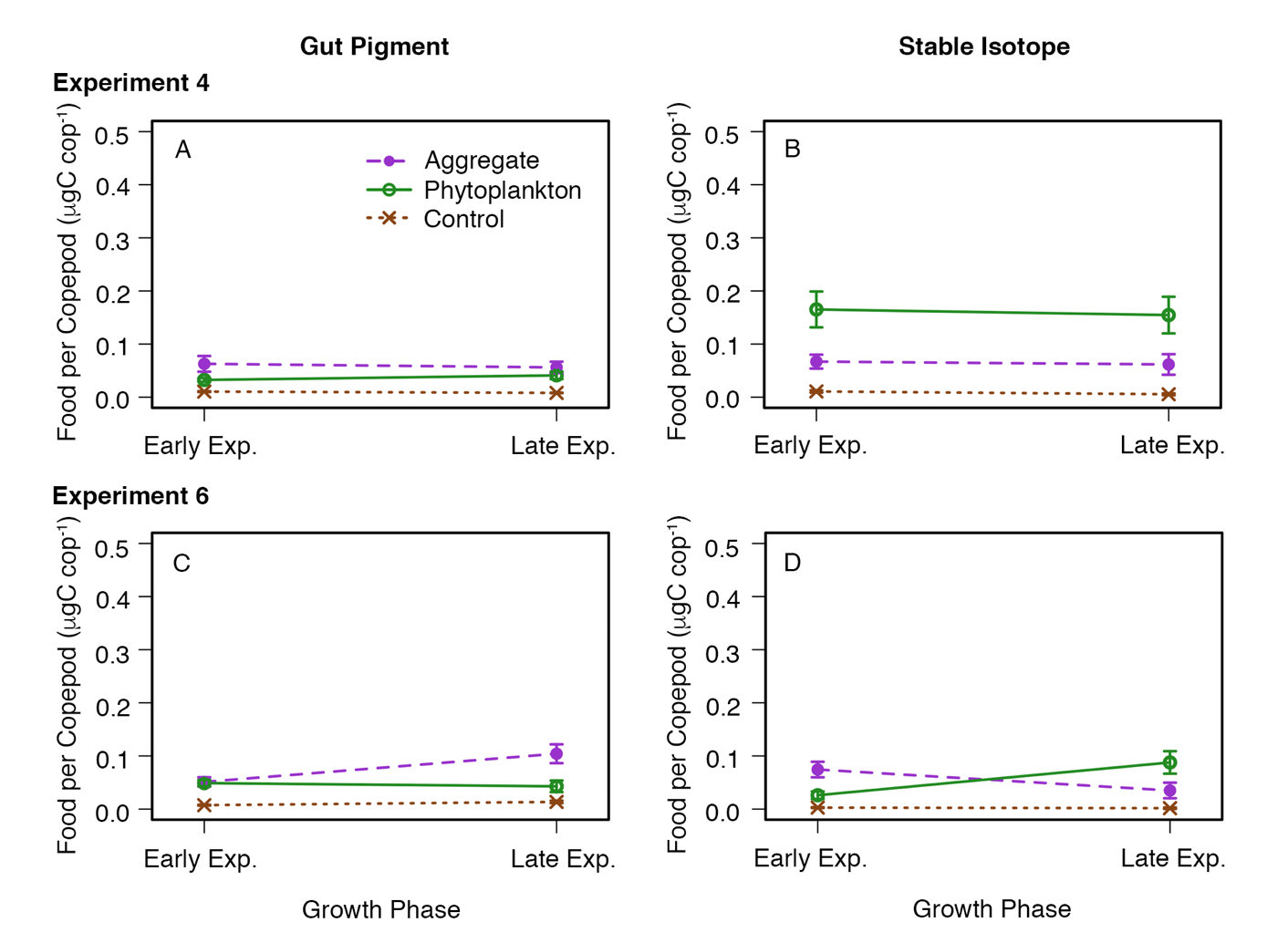


Supplementary Figure S2. Interaction plots of food per copepod versus growth phase (on x-axis) and treatment (shown in different colors and symbols) as calculated from gut pigment data (first column) and stable isotope analysis data (second column) for the two experiments using the phytoplankton species *S. maranoi*: (A and B) Experiment 4, (C and D) Experiment 6. Error bars represent standard error.


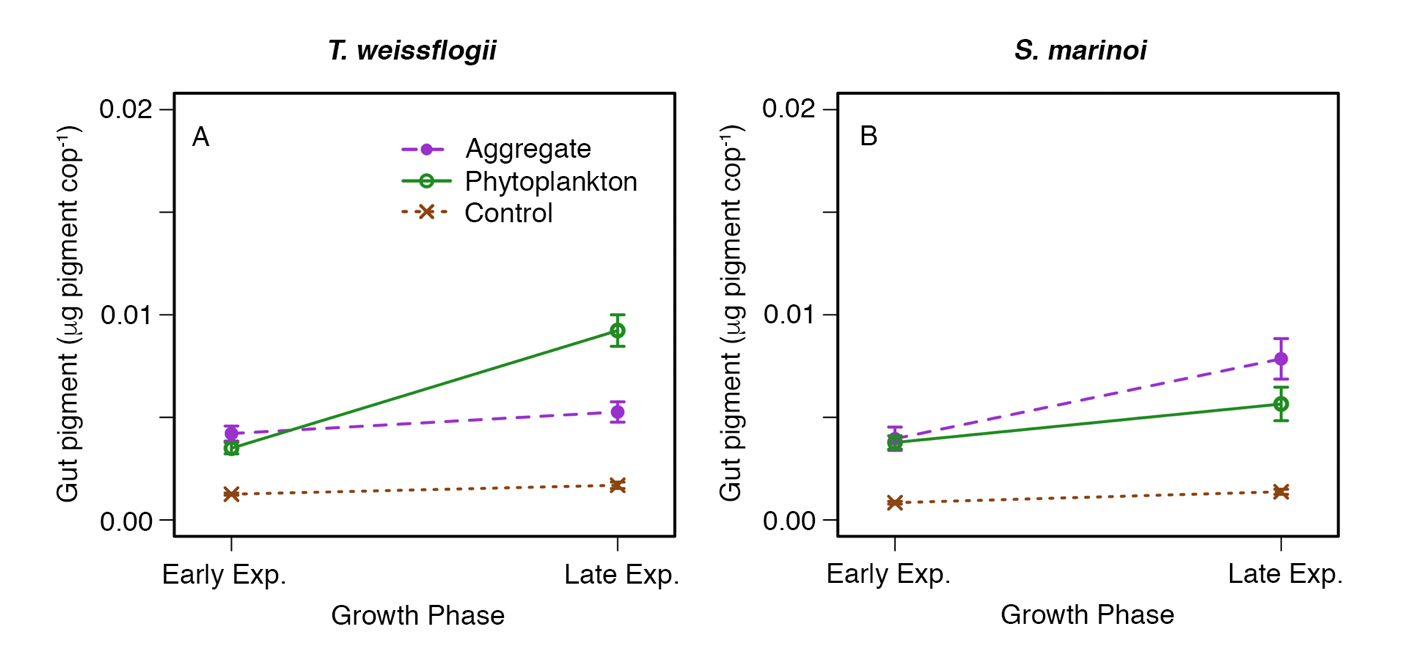


Supplementary Figure S3. Interaction plots of gut pigment versus growth phase (on x-axis) and treatment (shown in different colors and symbols) pooled for the four experiments using *T. weissflogii* (A), and the two experiments using the phytoplankton species *S. marinoi* (B). Error bars represent standard error. Two-way mixed-effect ANOVA tests for (A) show significant effect of treatment (p < 0.001), significant effect of growth phase (p < 0.001), and a significant interaction effect (p < 0.001). Two-way mixed-effect ANOVA tests for (B) show significant effect of treatment (p < 0.001), significant effect of growth phase (p < 0.001), and a significant interaction effect (p = 0.032).
